# CERVICAL ESOPHAGOGASTRIC ANASTOMOSIS FISTULA FOLLOWING ESOPHAGECTOMY: A PROBLEM WITH NO SOLUTION?

**DOI:** 10.1590/0102-672020210002e1619

**Published:** 2021-12-17

**Authors:** Flavio Roberto TAKEDA, Rubens Aissar SALLUM, Ivan CECCONELLO, Sérgio Carlos NAHAS

**Affiliations:** 1 Department of Gastroenterology. Digestive Surgery Division. Hospital das Clínicas, Faculty of Medicine, University of São Paulo, São Paulo, SP, Brazil

**Keywords:** Esofagectomia, Anastomose Cirúrgica, Fistula, Esofagectomia, Anastomose Cirúrgica, Fistula

The surgical treatment of esophageal cancer has evolved a lot in recent years. Forty years ago, the esophagectomy with lymphadenectomy technique in three dissection fields (cervical, thoracic and abdominal) proposed by Hiroshi Akiyama in 1981^1^ showed an increased survival of patients with esophageal neoplasia associated with extensive lymphadenectomy, currently representing the primary type of esophagectomy performed in the East. However, the surgical procedure’s morbidity rate was around 60%, with mortality of around 7%^1^. The advent of minimally invasive surgery in the late 1990s led to declining rates of postoperative complications, especially those of respiratory complications^6^. The publication of a prospective study comparing the hybrid minimally invasive esophagectomy technique with the conventional esophagectomy technique (thoracotomy and laparotomy) evidenced that the minimally invasive technique could have fewer postoperative complications without interfering with overall survival^4^.

In 1977, Professor Henrique Walter Pinotti proposed the transhiatal, also called transdiaphragmatic esophagectomy^5^. This esophagectomy procedure without thoracotomy showed a 40% decline in surgery-related complications^5^. More recently, performing the same procedure with the minimally invasive technique also showed a decrease of around 20%^7^.

Thus, in recent years, we observed an improvement in surgical results with acceptable rates of complications^10^. However, when evaluating the surgical results, transthoracic esophagectomy shows more extensive lymphadenectomy related to a more significant number of resected lymph nodes. In the past, this would translate into a direct increase in patient survival. Nowadays, with the advent of neoadjuvant therapies, the need for extensive lymphadenectomy has become very debatable in the literature. Transthoracic access allows resection^1^ of lymph nodes with a median of around 30, while conventional transhiatal access^7^ reaches 20 lymph nodes and laparoscopic transhiatal access 25 lympho nodes^3^.

However, despite the implementation of minimally invasive techniques, the rates of esophagogastric anastomosis fistula remain at around 10-15%^3^, regardless of the technique employed (manual, circular or linear stapling) and some surgical maneuvers (epiploplasty, pleural reconstruction, and use of surgical glue)^2^.

Recently, we proposed a surgical standardization following esophagectomy for revascularization of the gastric tube transposed by the posterior mediastinum using neck vessels (external jugular vein and transverse cervical artery), observing a proven improvement in local tissue perfusion, which reduced the occurrence of 10.4% of fistulas (control group) to no fistula (the group with microanastomosis)^8^. It is worth mentioning the technical difficulty of assessing tissue perfusion^9^. After all, the methodology employed should be technically easy to apply, as sensitive as possible, and reproducible, which is still a challenge. Probably, esophagogastric fistulas are not only related to tissue perfusion but also local factors such as, for example, hypertension caused by persistent postoperative cough and immunogenic factors.

Finally, cervical vascular microanastomosis (Supercharged Anastomosis For Esophagectomy - SAFE procedure) is a new perspective of reducing esophagogastric fistulas following esophagectomy. The next step will be to identify who would really benefit from SAFE, proven through randomized clinical trials.
